# Pulse oximetry

**DOI:** 10.1186/s13054-015-0984-8

**Published:** 2015-07-16

**Authors:** Amal Jubran

**Affiliations:** Division of Pulmonary and Critical Care Medicine, Edward Hines Jr. Veterans Affairs Hospital, 111N, 5000 South Fifth Avenue, Hines, IL 60141 USA; Loyola University of Chicago Stritch School of Medicine, 2160 South First Avenue, Maywood, IL 60153 USA

## Abstract

Pulse oximetry is universally used for monitoring patients in the critical care setting. This article updates the review on pulse oximetry that was published in 1999 in *Critical Care*. A summary of the recently developed multiwavelength pulse oximeters and their ability in detecting dyshemoglobins is provided. The impact of the latest signal processing techniques and reflectance technology on improving the performance of pulse oximeters during motion artifact and low perfusion conditions is critically examined. Finally, data regarding the effect of pulse oximetry on patient outcome are discussed.

## Introduction

Pulse oximetry is ubiquitously used for monitoring oxygenation in the critical care setting. By forewarning the clinicians about the presence of hypoxemia, pulse oximeters may lead to a quicker treatment of serious hypoxemia and possibly circumvent serious complications. In this review, I update the principles of pulse oximetry from my article in 1999 and discuss recent technological advances that have been developed to enhance the accuracy and clinical applications of this monitoring technique [1]. Finally, available studies evaluating the impact of pulse oximetry on patient outcome will also be reviewed.

## Principles of pulse oximetry

**Fig. 1:**
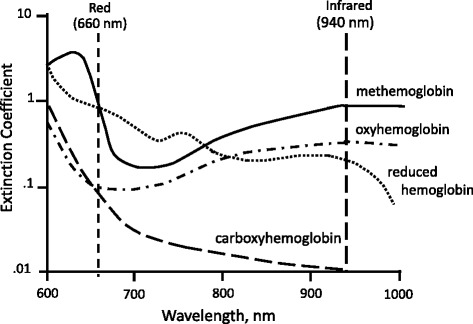
Transmitted light absorbance spectra of four hemoglobin species: oxyhemoglobin, reduced hemoglobin, carboxyhemoglobin, and methemoglobin

**Fig. 2:**
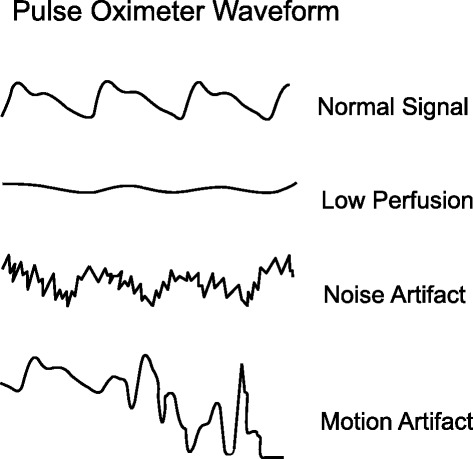
Common pulsatile signals on a pulse oximeter. (Top panel) Normal signal showing the sharp waveform with a clear dicrotic notch. (Second panel) Pulsatile signal during low perfusion showing a typical sine wave. (Third panel) Pulsatile signal with superimposed noise artifact giving a jagged appearance. (Bottom panel) Pulsatile signal during motion artifact showing an erratic waveform. Reprinted with permission from BioMed Central Ltd [1]

The technique of pulse oximetry has been previously described [1]. Using spectrophotometric methodology, pulse oximetry measures oxygen saturation by illuminating the skin and measuring changes in light absorption of oxygenated (oxyhemoglobin) and deoxygenated blood (reduced hemoglobin) using two light wavelengths: 660 nm (red) and 940 nm (infrared) [1,2] (Fig. 1). The ratio of absorbance at these wavelengths is calculated and calibrated against direct measurements of arterial oxygen saturation (SaO_2_) to establish the pulse oximeter’s measure of arterial saturation (SpO_2_). The waveform, which is available on most pulse oximeters, assists clinicians in distinguishing an artifact from the true signal (Fig. 2).

## Accuracy

**Fig. 3:**
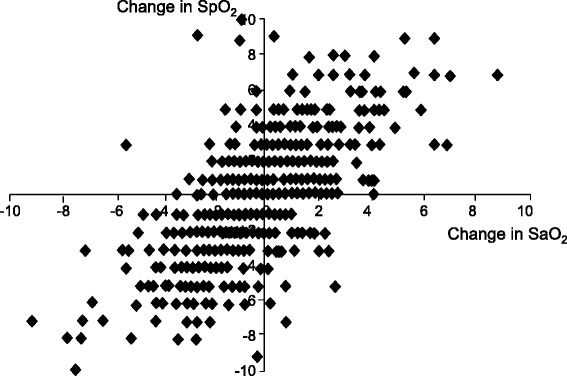
Changes in oxygen saturation measured by pulse oximetry (SpO_2_) compared with arterial oxygen saturation measured by a CO-oximeter (SaO_2_) in critically ill patients. The pulse oximeter consistently overestimated the actual changes of SaO_2_. Reprinted with permission from BioMed Central Ltd [8]

In critically ill patients with SaO_2_ values of 90 % or higher, the mean difference between SpO_2_ and SaO_2_ (that is, bias) measured by a reference standard (CO-oximeter) is less than 2 %; the standard deviation of the differences between the two measurements (that is, precision) is less than 3 % [3–5]. The bias and precision of pulse oximetry readings, however, worsen when SaO_2_ is lower than 90 % [6,7]. Although pulse oximetry is accurate in reflecting one-point measurements of SaO_2_, it does not reliably predict changes in SaO_2_, particularly in intensive care unit (ICU) patients [5,8] (Fig. 3).

The conventional pulse oximeters use transmission sensors in which the light emitter and detector are on opposing surfaces of the tissue bed. These sensors are suitable for use on the finger, toe, or earlobe; when tested under conditions of low perfusion, finger probes performed better than other probes [9]. Recently, pulse oximeter probes that use reflectance technology have been developed for placement on the forehead [10]. The reflectance sensor has emitter and detector components adjacent to one another, so oxygen saturation is estimated from back-scattered light rather than transmitted light. In critically ill patients with low perfusion, the bias and precision between SpO_2_ and SaO_2_ were lower for the forehead reflectance probe than for the finger probe [11,12]. The superiority of forehead reflectance probes over conventional digital probes, however, was not observed in patients with acute respiratory distress syndrome (ARDS) during a positive end-expiratory pressure (PEEP) recruitment maneuver [13].

**Fig. 4:**
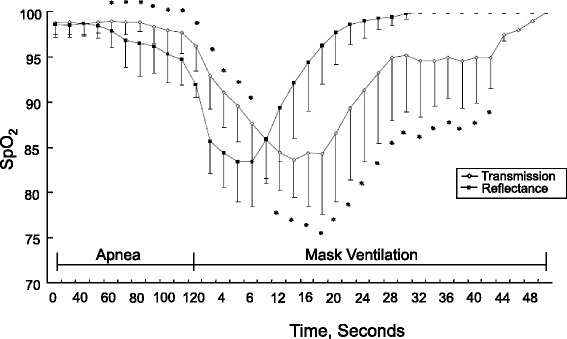
Oxygen saturation measured with pulse oximetry (SpO_2_) using transmittance finger probe (diamond) and reflectance forehead probe (squares) during apnea and mask ventilation with 100% O_2_. The reflectance probe showed faster responses than the transmission probe at every measurement point. *P < 0.05 between the two groups. Reprinted with permission from Wiley [16]

The response time of conventional oximeter probes varies; ear probes respond quicker to a change in O_2_ saturation than finger probes [14,15]. A recent study compared the response time of the conventional finger probe with the reflectance forehead probe in patients undergoing general anesthesia [16] (Fig. 4). The lengths of time it took to detect a decrease in SpO_2_ to 90 % after apnea was induced (desaturation response time) were 94 seconds for the forehead probe and 100 seconds for the finger probe. After mask ventilation was started, the lengths of time it took to detect an increase in SpO_2_ to 100 % (re-saturation response time) were 23.2 seconds for the forehead probe and 28.9 seconds for the finger probes. The investigators speculated that the shorter response time with the reflectance forehead probe was most likely due to the location of the probe rather than to the workings of the reflectance technology. The forehead probe monitors O_2_ saturation from the supraorbital artery in which blood flow is abundant and is less likely to be affected by vasoconstriction than is a peripheral artery [17].

## Limitations

Oximeters have limitations which may result in erroneous readings [15] (Table 1). Because of the sigmoid shape of the oxyhemoglobin dissociation curve, oximetry may not detect hypoxemia in patients with high arterial oxygen tension (PaO_2_) levels [1,18].

**Table 1:** Limitations of pulse oximetry

Shape of oxygen dissociation curve	
Dyshemoglobins	
- Carboxyhemoglobin	
- Methemoglobin	
Dyes	
Low perfusion state	
Skin pigmentation	
Anemia	
Nail polish	
Motion artifact	
Limited knowledge of the technique	

Conventional pulse oximeters can distinguish only two substances: reduced hemoglobin and oxyhemoglobin; it assumes that dyshemoglobins—such as carboxyhemoglobin (COHb) and methemoglobin (MetHb)—are absent (Fig. 1). Studies showed that the presence of elevated levels of COHb and MetHb could affect the accuracy of SpO_2_ readings [1,19]. Accordingly, multiwavelength oximeters that are capable of estimating blood levels of COHb and MetHb have recently been designed [20]. In healthy volunteers, the accuracy of a multiwavelength oximeter (Masimo Rainbow-SET Rad-57 Pulse CO-oximeter; Masimo Corporation, Irvine, CA, USA) in measuring dyshemoglobins was evaluated by inducing carboxyhemoglobinemia (levels range from 0 % to 15 %) and methemoglobinemia (levels range from 0 % to 12 %) [20]. Bias between COHb levels measured with the pulse CO-oximeter and COHb levels measured with the laboratory CO-oximeter (standard method) was −1.22 %; the corresponding precision was 2.19 %. Bias ± precision of MetHB measured with the pulse CO-oximeter and MetHb measured with the laboratory CO-oximeter was 0.0 % ± 0.45 %. The accuracy of pulse CO-oximeters in measuring COHb levels was also assessed during hypoxia [21]. In 12 healthy volunteers, the pulse CO-oximeter was accurate in measuring COHb at an SaO_2_ of less than 95 % (bias of −0.7 % and precision of 4.0 %); however, when the SaO_2_ dropped below 85%, the pulse CO-oximeter was unable to measure COHb levels. In patients evaluated in the emergency department with suspected carbon monoxide poisoning, the bias between pulse CO-oximetric measurement of COHb and laboratory CO-oximetric measurement of COHb was less than 3 % [22,23]. The limits of agreement between the measurements, however, were large (−11.6 % to 14.14 %) [23], leading some authors to conclude that these new pulse CO-oximeters may not be used interchangeably with standard laboratory measurements of COHb [22–24].

**Fig. 5:**
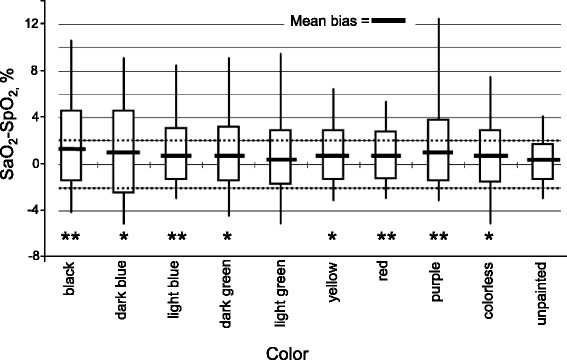
Bias of O_2_ saturation pulse oximetry (SpO_2_) and arterial O_2_ saturation (SaO_2_) of various nail polish colors in critically ill patients. Thick horizontal lines represent mean bias, the whiskers represent maximum and minimum bias; the bottom and top of the boxes represent the first and third quartiles. *P < 0.05 ,**P < 0.01 when compared with arterial oxygen saturation. Reprinted with permission from Elsevier Inc. [29]

Inaccurate readings with pulse oximetry have been reported with intravenous dyes used for diagnostic purposes, low perfusion states (that is, low cardiac output, vasoconstriction, and hypothermia), pigmented subjects and in patients with sickle cell anemia [1,6,25,26]. Because the two wavelengths (660 and 940 nm) that pulse oximeters use to measure SpO_2_ can be produced by various ambient light sources, the presence of such sources could produce false SpO_2_ readings. To test the accuracy of pulse oximetry in the presence of ambient light, Fluck and colleagues [27] performed a randomized controlled trial in healthy subjects in which SpO_2_ measurements were obtained in a photographic darkroom under five separate light sources: quartz-halogen, infrared, incandescent, fluorescent, and bilirubin light [27]. The largest difference in SpO_2_ between the control condition (that is, complete darkness) and any of the five light sources was less than 5%. Nail polish can interfere with pulse oximetry readings [28]. In 50 critically ill patients requiring mechanical ventilation, Hinkelbein and colleagues [29] found that the mean difference between SpO_2_ and SaO_2_ was greatest for black (+1.6 % ± 3.0 %), purple (+1.2 % ± 2.6 %), and dark blue (+1.1 % ± 3.5 %) nail polish; limits of agreement ranged from 6 % (unpainted fingernail) to 14.4 % (dark blue) (Fig. 5). Rotating the oximeter finger probe by 90 ° did not eliminate the error induced with nail polish.

Motion artifact is considered an important cause of error and false alarms [30–33]. In the 1990s, several signal processing techniques were incorporated in pulse oximeters in an attempt to reduce motion artifact [34–38]. One such technique is Masimo signal extraction technology (SET™) [39]. During motion and hypoxia, the Masimo SET oximeter performed better than the Agilent Viridia 24C (Agilent Technologies, Santa Clara, CA, USA), the Datex-Ohmeda 3740 (Datex-Ohmeda, Madison, WI, USA), and the Nellcor N-395 (Covidien Corporation, Dublin, Ireland) oximeters [34].

The knowledge about pulse oximetry among clinicians continues to be limited. When 551 critical care nurses were recently interviewed, 37 % of them did not know that oximeters were more likely to be inaccurate during patient motion, 15 % did not know that poor signal quality can produce inaccurate readings, and 30 % considered that SpO_2_ readings could be used in lieu of arterial blood gas samples when managing ICU patients [40].

## Clinical applications

Pulse oximetry can provide an early warning of hypoxemia [41,42]. In the largest randomized trial involving more than 20,000 perioperative patients, rates of incidence of hypoxemia (SpO_2_ of less than 90 %) were 7.9 % in patients who were monitored with pulse oximetry and only 0.4 % in patients without an oximeter [43]. The anesthesiologists reported that oximetry led to a change in therapy on at least one occasion in up to 17 % of the patients. Using 95,407 electronically recorded pulse oximetry measurements from patients who underwent non-cardiac surgery at two hospitals, Ehrenfeld and colleagues [44] reported that during the intraoperative period, 6.8 % of patients had a hypoxemic event (SpO_2_ of less than 90) and 3.5 % of patients had a severe hypoxemic event (SpO_2_ of not more than 85 %) lasting more than 2 minutes. Hypoxemic events occurred mostly during the induction or emergent phase of anesthesia; these time periods are consistent with the clinical view that anesthesia-transitional states are high-risk periods for hypoxemia [45]. In patients undergoing gastric bypass surgery, continuous monitoring of SpO_2_ revealed that episodic hypoxemia (SpO_2_ of less than 90 % for at least 30 seconds) occurred in all patients. For each patient, desaturation lasted as long as 21 ± 15 minutes [46].

Pulse oximetry has been shown to be reliable in titrating the fractional inspired oxygen concentration (F_I_O_2_) in patients requiring mechanical ventilation; aiming for an SpO_2_ of 92 % is reasonable for ensuring satisfactory oxygenation in Caucasian patients [6]. To determine whether the ratio of SpO_2_ to F_I_O_2_ (S/F) can be used as a surrogate for the ratio of PaO_2_ to F_I_O_2_ (P/F), SpO_2_ and PaO_2_ data from 1,074 patients with acute lung injury or ARDS who were enrolled in two large clinical trials were compared [47]. An S/F ratio of 235 predicted a P/F ratio of 200 (oxygenation criterion for ARDS), a sensitivity of 0.85, and a specificity of 0.85. An S/F ratio of 310 reflected a P/F ratio of 300 (oxygenation criterion for acute lung injury), a sensitivity of 0.91, and a specificity of 0.56. In patients undergoing surgery, the S/F ratio was shown to be a reliable proxy for the P/F ratio (correlation coefficient (r) of 0.46), especially in those patients requiring PEEP levels of greater than 9 cm H_2_O (r = 0.68) and those patients with a P/F ratio of 300 or less (r = 0.61) [48]. In the ICU, the S/F ratio can also be a surrogate measure for the P/F ratio when calculating the sequential organ failure assessment score, which measures the severity of organ dysfunction in critically ill patients [49].

## Cost-effectiveness

Studies have shown that the presence of pulse oximetry may reduce the number of arterial blood gas samples obtained in the ICU and in the emergency department [50,51]. However, the lack of incorporating explicit guidelines for the appropriate use of pulse oximetry may lessen the cost-effectiveness of pulse oximetry in the ICU [1].

## Effect on outcome

To date, the largest randomized controlled trial that has evaluated the impact of pulse oximetry on outcome was the study by Moller and colleagues [43] in 20,802 surgical patients. Although myocardial ischemia occurred less frequently in the oximetry than the control group, the numbers of post-operative complications and hospital deaths were similar in the two groups [43].

In a more recent randomized study in 1,219 post-operative patients, Ochroch and colleagues [52] assessed the impact of pulse oximetry on the rate of transfer to the ICU from a post-surgical care floor. Upon admission to the study floor, patients were randomly assigned to receive monitoring with a pulse oximeter either continuously (n = 589) (oximeter group) or intermittently (n = 630) according to clinical needs as judged by a nurse or a physician (control group). The percentages of patients transferred to the ICU were similar in the oximeter group and the control group (6.7 % versus 8.5 %). A lower rate of ICU transfers for pulmonary complications was noted in the oximeter group. For those patients who required ICU transfer, the estimated cost from enrollment to completion of the study was less in the oximeter group ($15,481) than in the control group ($18,713) despite the older age and higher comorbidity of the former. The authors speculate that reduction in pulmonary transfers to the ICU may be due to the earlier recognition and treatment of post-operative pulmonary complications.

The lack of demonstrable benefit of pulse oximetry on outcome in clinical trials may be due to the signal-to-noise ratio [41,53]. Because the outcome under evaluation (readmission to the ICU, myocardial infarction, or death) is rare, a huge number of patients are needed to show a reduction in these events [41]. To demonstrate a reduction in complications in the study by Moller and colleagues, for example, a 23-fold increase in enrollment would have been required [41,53].

The fact that randomized trials failed to demonstrate that routine monitoring with pulse oximetry improved patient outcome has not stopped anesthesiologists from using pulse oximeters [53,54]. When surveyed, 94 % of the anesthesiologists in the study by Moller and colleagues [43] considered the pulse oximeters to be helpful in guiding clinical management. They believed that maintaining oxygenation within the physiologic limits with the help of pulse oximetry might help avert irreversible injury. It is this perspective that has made pulse oximetry a crucial part of standard of care despite the absence of proven efficacy [41].

## Conclusions

Pulse oximetry is universally used for monitoring respiratory status of patients in the ICU. Recent advances in signal analysis and reflectance technology have improved the performance of pulse oximeters under conditions of motion artifact and low perfusion. Multiwavelength oximeters may prove to be useful in detecting dyshemoglobinemia. Monitoring with pulse oximetry continues to be a critical component of standard of care of critically ill patients despite the paucity of data that such devices improve outcome.

## Abbreviations

ARDS: acute respiratory distress syndrome
COHb: carboxyhemoglobin
F_I_O_2_: fractional inspired oxygen concentration
ICU: intensive care unit
MetHb: methemoglobin
PaO_2_: arterial oxygen tension
PEEP: positive end-expiratory pressure
P/F: PaO_2_-to-F_I_O_2_ ratio
r: correlation coefficient
SaO_2_: arterial oxygen saturation
SET: signal extraction technology
S/F: SpO_2_-to-F_I_O_2_ ratio
SpO_2_: oxygen saturation measured by pulse oximetry
